# Combination of azathioprine and prednisolone as a treatment for meningoencephalomyelitis of unknown origin in dogs: 54 cases

**DOI:** 10.1093/jvimsj/aalaf002

**Published:** 2026-01-21

**Authors:** Solene Diop, Nicolas Van Caenegem, Thibaut Troupel, Vincent Mayousse, Aurélien Jeandel, Stéphane Blot

**Affiliations:** École Nationale Vétérinaire d'Alfort, 94700 Maisons-Alfort, France; Université Paris-Est Créteil, INSERM, U955 IMRB “Biology of the Neuromuscular System” Team, 94700 Maisons-Alfort, France; Clinique Vétérinaire AniCura TRIOVet, 35000 Rennes, France; Centre Hospitalier Vétérinaire Anicura Pommery, 51100 Reims, France; Centre Hospitalier Vétérinaire des Cordeliers, 77100 Meaux, France; Centre Hospitalier Vétérinaire Anicura Pommery, 51100 Reims, France; École Nationale Vétérinaire d'Alfort, 94700 Maisons-Alfort, France; Université Paris-Est Créteil, INSERM, U955 IMRB “Biology of the Neuromuscular System” Team, 94700 Maisons-Alfort, France

**Keywords:** canine, meningoencephalitis, immunomodulation, immune-mediated diseases

## Abstract

**Background:**

Information regarding efficacy of combination of azathioprine and prednisolone in treating meningoencephalomyelitis of unknown origin (MUO) in dogs is limited.

**Hypothesis/Objectives:**

To report median survival time in dogs with MUO treated with a combination of azathioprine and prednisolone and assess its tolerability.

**Animals:**

Ninety-two dogs diagnosed with MUO between January 2011 and December 2022 that received azathioprine and prednisolone.

**Methods:**

A retrospective medical record review was conducted. Diagnosis of MUO was based on clinical signs, magnetic resonance imaging features, and cerebrospinal fluid analysis.

**Results:**

Median survival time was 1289 days (range: 30-3356). Median survival time did not differ significantly between dogs that presented with seizures (*n* = 12; 308 days) and those without seizures (1289 days; *P* = .74), nor between dogs that experienced relapses (*n* = 20; 1153 days) and those that did not (1697 days; *P* = .58). Myelosuppression occurred in 10 dogs and hepatotoxicity in 1 dog. In 9 dogs, adverse effects resolved with drug discontinuation or dose reduction. One dog died from hepatic necrosis and 1 from myelosuppression.

**Conclusions and clinical importance:**

Combination of azathioprine and prednisolone is associated with long median survival times for dogs with MUO in this study. Adverse drug reactions were reversible in most cases but can be fatal in some. Therefore, hematological values and hepatic variables should be monitored.

## Introduction

Meningoencephalomyelitis of unknown origin (MUO) is a relatively common neurological inflammatory disorder in dogs; its prevalence is estimated at around 5%-25% among all central nervous system (CNS) disorders in dogs.^1^ The term of MUO includes a variety of entities, including necrotizing leucoencephalitis (NLE), necrotizing meningoencephalomyelitis (NME), and granulomatous meningoencephalitis (GME).^2^ Young to middle-aged toy and terrier breeds have a greater likelihood of developing GME,^3^ although this disease can occur in any breed. NME often affects Pugs, Chihuahuas, and Maltese dogs, whereas NLE is most frequently seen in Yorkshire Terriers and French Bulldogs.^4-9^ In some dogs, multiple subtypes of MUO have been identified, supporting the concept of a unique disease entity with pleomorphic disease expression.^10^ The exact pathogenesis remains unclear. Previous studies have indicated that the occurrence of seizures could serve as a negative prognostic factor. Although less evidence exists regarding relapses, they are suspected to be associated with a poor prognosis.

This disease is associated with high fatality, up to 26% in the 7 days after diagnosis.^11^ Treatment plan of MUO varies but often rely on administration of corticosteroids alone, or on a combination of corticosteroids with 1 or several immunosuppressive drugs. The combination is thought to enhance immunosuppression and to limit adverse effects of administration of high-dose corticosteroids.^12^ Cytosine arabinoside,^13^^,^^14^ ciclosporine,^15-17^ azathioprine,^18^ lomustine, procarbazine, mycophenolate mofetil, and leflunomide are sometimes administered.^19^ Azathioprine effectiveness in treating immune-mediated diseases has been studied for various conditions, for example, immune-mediated hemolytic anemia,^20^ and steroid-responsive meningitis arteritis.^21^ Azathioprine is also widely available, easily dispensed (as a non-cancerigenous, non-mutagenic, or reprotoxic substance, in contrast with aracytine) and is affordable (in contrast with ciclosporine).

To the authors’ knowledge, there is only 1 study to date to evaluate the combination of azathioprine and prednisolone in dogs with MUO, with long median survival times when compared to other immunosuppressive drugs.^18^

The aim of this study was to determine the median survival time of dogs with MUO treated with the combination of prednisolone and azathioprine, and to evaluate the nature and prevalence of adverse effects. The secondary goal of this study was to determine if seizures or relapses could be associated with different median survival times, as identified in previous studies.^18^

## Material and methods

### Inclusion criteria and data collection

Medical records of dogs diagnosed with MUO at Ecole nationale vétérinaire d'Alfort that received an association of azathioprine and prednisolone as first-line treatment between January 2011 and December 2022 were retrospectively reviewed, using the medical database CLOVIS 4D v13. Diagnosis of MUO was presumptive and based on formerly established guidelines.^22^ The diagnosis relied on the following criteria:

1. Neurological examination that was consistent with MUO.
2. Magnetic resonance imaging (MRI) findings that were most consistent with non-infectious inflammatory changes of the CNS, demonstrated by either single, multifocal, or diffuse intra-axial hyperintense lesions on T2W images, and, if CSF analysis was performed, CSF pleocytosis (total nucleated cell count [TNCC] > 5 nucleated cells/L; erythrocyte count < 4000 cells/L) with >50% mononuclear cells; and, if available, negative test results for infectious diseases.

Cases of optic neuritis without any other neurological deficits were only included if they exhibited multifocal or diffuse intra-axial hyperintense lesions on T2W MRI. Dogs with incomplete medical records were excluded from the study. Dogs that began treatment with azathioprine but later received an additional immunosuppressive agent were censored at the time the second treatment was initiated.

All the following information was collected from medical records: signalment, body weight, clinical signs at first presentation, the interval between clinical sign onset and initial presentation, neuroanatomical lesion localization, MRI imaging findings, CSF analysis, detail of treatment protocol, and, when reported, adverse effects attributed to treatment.

### Diagnostic modalities

Magnetic resonance imaging was performed under general anesthesia on all animals, with either a 0.2 T MRI (Signa Profile, 0.2 T, General Electric) or a 1.5 T Magnet (Signa HD23 optima 1·5 T, General Electric).

The MRI sequences included at minimum T1- and T2-weighted sequences, fluid-attenuated inversion recovery sequences for brain imaging, fat saturation T1 sequences for spine imaging, and T1-weighted images after administration of intravenous gadolinium injection.

Cerebrospinal fluid was collected under anesthesia at the level of the cerebellomedullary cistern or the lumbar cistern, depending on the localization of the lesion. Pleocytosis was defined as TNCC higher than 5 cells per microliter. Protein level was defined as increased if it was higher than 0.25 g/L for cerebellomedullary cistern collection, and higher than 0.45 g/L for the lumbar cistern.

### Treatment

All dogs received prednisolone and azathioprine. Dose, duration of treatment, and additional treatment (including antiepileptic drugs and proton pump inhibitors) were retrieved from medical records. Medical records were also reviewed to identify potential adverse effects of treatment. Adverse effects attributed to either prednisolone or azathioprine were recorded.

### Outcome

Relapse was defined as deterioration in neurological status under treatment or after discontinuation of treatment. If MRI examination or CSF tap were repeated at the onset of relapse, information was recorded.

Cause of death was attributed to MUO if death resulted from the neurological disease or the necessity for euthanasia due to the progression of the disease. Other causes of death were not considered to be associated with MUO. All other dogs who were lost to follow-up had a final follow-up date recorded based on their last recorded visit or communication. These cases were censored in the survival analysis.

### Statistical analysis

Because all variables were non-normally distributed, data are presented as medians and IQRs. Median survival time was calculated for all dogs using Kaplan–Meier survival curves via R-derived software (version 3.6.3, R Foundation for Statistical Computing). Log-rank tests were performed to compare dogs that presented seizures with dogs that did not and dogs that presented relapse with dogs that did not. To better reflect the real-world median survival time, we also identified dogs that were intended to receive the treatment but died before its administration and calculated the median survival time using an intention-to-treat approach. As this study did not include a sample size calculation, these analyses are presented as exploratory and intended to generate hypotheses for future research.

## Results

### Signalment and clinical signs

**Figure 1:**
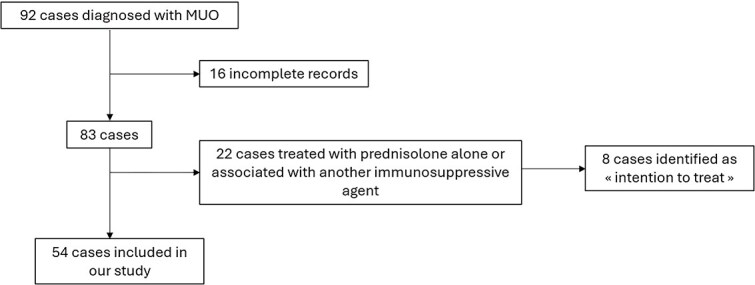
Flow chart of inclusion process in the study.

Ninety-two dogs were diagnosed with MUO during the study period. Fifty-four dogs met the inclusion criteria. We identified 8 dogs that were initially slated to receive azathioprine, but died too soon to receive the treatment (Figure 1). Affected breeds included Yorkshire Terrier (*n* = 18), French Bulldog (*n* = 7), Chihuahua (*n* = 6), Maltese (*n* = 4), crossbreed (*n* = 3), Spitz (*n* = 2), Cavalier King Charles (*n* = 2), Jack Russell Terrier (*n* = 1), Pug (*n* = 1), Staffordshire Bull Terrier (*n* = 1), Shetland Sheepdog (*n* = 1), Poodle (*n* = 1), English Bulldog (*n* = 1), Border Collie (*n* = 1), Miniature Pinscher (*n* = 1), Dutch Shepherd (*n* = 1), West Highland White Terrier (*n* = 1), Australian Shepherd (*n* = 1), and Beauceron (*n* = 1). Median weight was 4.6 kg (IQR, [4.75; 19.5]). Key information regarding cases are summarized in Table S1.

Dogs exhibited clinical signs at a median age of 48 months (IQR [25; 68]). The sex ratio was 40% female (*n* = 22) to 60% male (*n* = 32). Median time of presentation after onset of clinical signs was 14 days (range 1-730).

Anatomical localization of clinical signs was prosencephalon in 23 dogs, brainstem in 6 dogs, spinal cord in 5 dogs, cerebellum in 1 dog, and multifocal in 12 dogs. In dogs with multifocal localization, 2 dogs presented both signs of myelopathy cord and encephalopathy. Seven dogs presented loss of vision with absent or reduced pupillary light reflexes as only clinical signs, despite presenting multifocal intra-axial lesions on MRI.

### Diagnostic modalities

Magnetic resonance imaging was available in 53 cases. Five dogs were diagnosed with a 0.2 T MRI; the rest of the dogs underwent MRI examination with a 1.5 T Magnet. The MRI study was normal for 6 cases (12%), and among them, 1 dog had received prednisolone before presentation. Lesions were multifocal for 49 cases (92%) and focal for only 4 cases (8%). All cases that were included with loss of vision as the only clinical sign, had multifocal lesions (involvement of optic tracts and forebrain). For 1 dog, MRI was not performed, but a computed tomography scan was performed under light sedation because the dog was too unstable to undergo general anesthesia. The computed tomography study was abnormal for this dog and showed a diffuse hypodense signal in the medio-dorsal part of the left parietal lobe, consistent with inflammatory lesions.

Cerebrospinal fluid analysis was performed in 52 cases.

In 1 case, intracranial hypertension was suspected, which prevented sampling. In the other case, sampling failed. For 2 dogs, samples presented severe blood contamination that prevented analysis. Cerebrospinal fluid analysis was normal for 11 dogs (21%). Monocytic pleocytosis was identified in 26 dogs, 4 dogs presented mixed neutrophilic and lymphocytic pleocytosis. Mixed pleocytosis with lymphocytes and eosinophils was present in 1 case. Median TNCC at diagnosis was 12 cells/μL (IQR [5; 36.5]). Four dogs presented albuminocytological dissociation.

C-reactive protein was measured in 15 dogs and was elevated (>10 mg/L) in 7 cases (median, 36 mg/L; IQR [19.55; 53.8]).

Infectious agents testing for Canine distemper virus, *Toxoplasma gondii*, and *Neospora caninum*, was performed by polymerase chain reaction or by serologic assays. These tests were negative for the tested dogs (*n* = 37).

All cases with optic tract implication had fundus examination. Two dogs displayed signs of papillitis.

### Treatment

Because of the long study period, treatment protocols varied from 1 dog to another. Treatment usually consists of prednisolone at immunosuppressive dose (≥2 mg/kg/day) for the first 2-4 weeks, then is progressively tapered down every 6-8 weeks. Azathioprine was initiated at the time of diagnosis—meaning in the days after the MRI for the dogs without infectious testing, and usually 7-10 days after the MRI for the dogs with infectious testing. Azathioprine was either maintained at the initial dose of 2 mg/kg/day for the complete duration of treatment (*n* = 43) or was progressively tapered down after 2 weeks after introduction (*n* = 11).

All 12 dogs that presented seizures received anti-epileptic treatment (phenobarbital, levetiracetam, or both).

### Outcome

**Figure 2:**
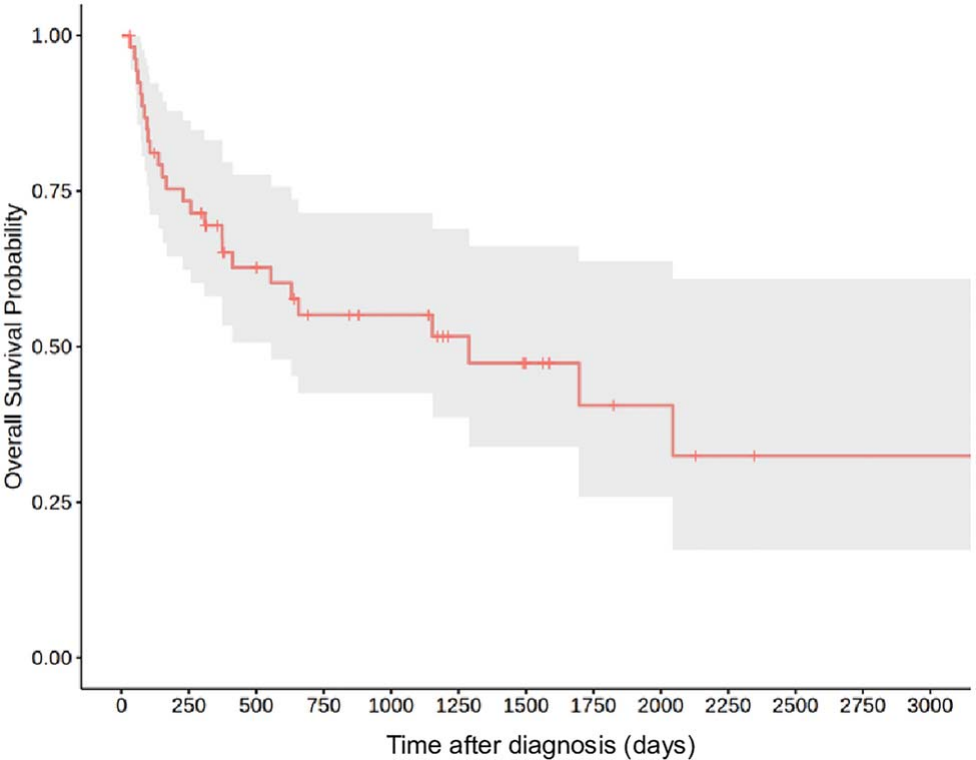
Kaplan–Meier curve for the 54 dogs treated with azathioprine and prednisolone the gray area represents the 95% CI.

**Figure 3:**
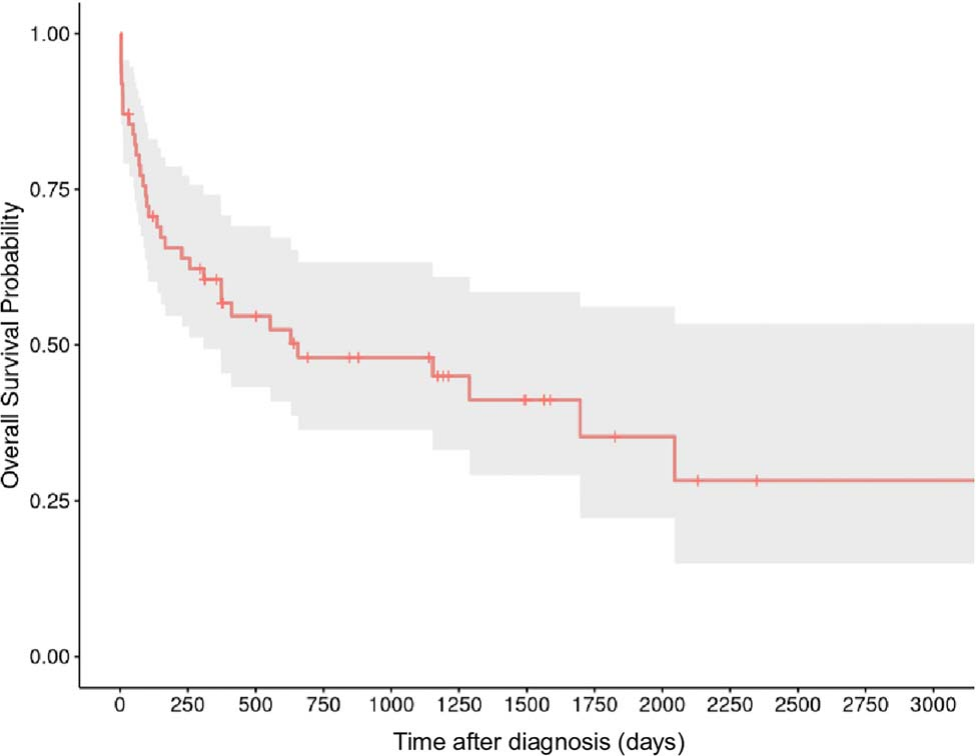
Kaplan–Meier curve for the 54 dogs treated with azathioprine and prednisolone and the 8 dogs that were identified in intention to treat strategy (received only prednisolone and died before receiving azathioprine). The gray area represents the 95% CI.

Median survival time was 1289 days (range: 30-3356 days, IQR: 228-3216 days; Figure 2). In Figure 3, we included the 8 dogs in intention-to-treat strategy, leading to shorter survival times: 656 days (range: 3-3356 days, IQR: 94-3356 days). However, to evaluate relapse and seizure on prognosis, we excluded these cases.

**Figure 4:**
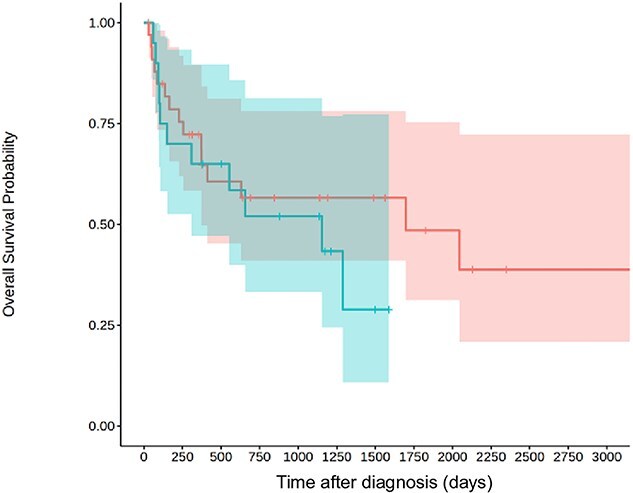
Kaplan–Meier curve for dogs who presented relapses under treatment (blue, *n* = 20) and dogs who did not presented relapses (red, *n* = 34). The 95% CIs are represented in blue and red, respectively.

Relapse under treatment occurred in 20 dogs. Median survival time was not significantly different for dogs that presented relapses (1153 days; IQR: 105-? days) and dogs that did not present relapses (1697 days; IQR: 256-3356 days; *P* = .58; Figure 4)*.*

**Figure 5:**
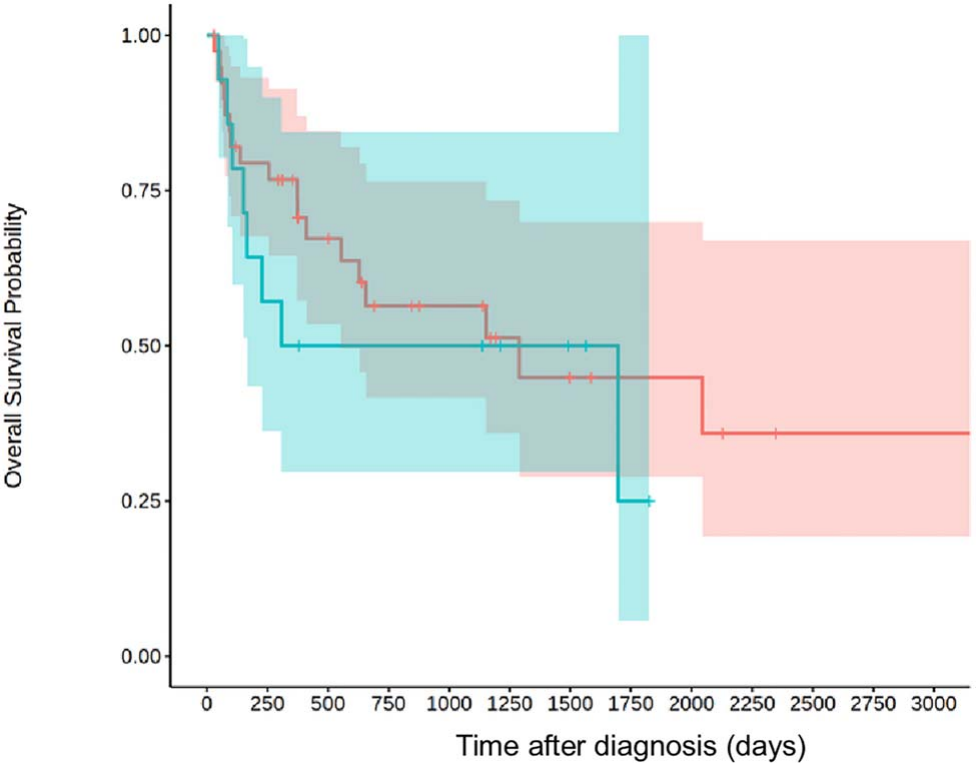
Kaplan–Meier curve for dogs who presented seizures (blue, *n* = 14) and dogs who did not presented seizures (red, *n* = 30). The 95% CIs are represented in blue and red, respectively.

Median survival time was not significantly different for dogs that presented seizures (308 days; IQR: 151-1697 days) and dogs that did not present seizures (1289 days; IQR: 373-3356 days; *P* = .74; Figure 5).

Six dogs (11%) entered complete remission, even after discontinuation of azathioprine and prednisolone. For dogs that achieved remission, median time between interruption of treatment and last follow up was 596.5 days (range 172-2634).

### Adverse effects

Blood tests were performed every 6-8 weeks. Adverse effects attributed to azathioprine were identified in 11 dogs (20%). Ten dogs presented signs of myelosuppression, with either neutropenia and thrombopenia (*n* = 3), non-regenerative normocytic normochromic anemia (*n* = 6), or both (*n* = 1). Anemia was moderate for most cases (median hematocrit: 31%), but was severe in 1 case (hematocrit: 11%). In all cases but the last, anemia resolved upon dose reduction or discontinuation of azathioprine. In the last case, severe anemia persisted and worsened over the course of 10 days, even after discontinuation of treatment. Other causes of non-regenerative anemia were ruled out. The dog eventually died due to this adverse effect of treatment. Median time of onset of adverse effects was 140 days (range 32-700 days, IQR: 87-334 days; Figure S1).

One dog in our study presented severe acute hepatic failure 9 months after initiation of azathioprine. Infectious diseases were investigated, but tests failed to identify underlying cause. No toxic exposition other than azathioprine was reported. The dog died even after discontinuation of azathioprine. Histology of the liver was consistent with hepatocellular necrosis, suspected to be of toxic origin.

## Discussion

In this study, the combination of azathioprine and prednisolone was associated with long median survival times in dogs with MUO. The median survival time observed in our study was comparable to that reported in a previous study investigating the use of azathioprine in the treatment of MUO in dogs.^18^ Furthermore, the median survival time achieved with azathioprine in our study was comparable to or superior to the median survival times reported with alternative immunosuppressive drugs in previous studies.^13-15^^,^^22^^,^^23^ When using an intention-to-treat approach that includes dogs initially planned to receive azathioprine but who died prematurely, the median survival time was shorter and similar to the median survival times previously reported with prednisolone alone. The intention-to-treat strategy preserves the benefits of random assignment by including all dogs in the analysis, regardless of whether they received the treatment as planned or not. It is highly advised for comparative therapeutic studies as for observational ones. This raises the question of whether corticosteroids alone could be used in treating MUO, given the low proof of evidence of other treatment strategies.^12^^,^^24^

The second objective of our investigation was to assess the safety of azathioprine in treating MUO. One dog developed acute hepatocellular necrosis, which was potentially attributable to azathioprine, occurring approximately 9 months after initiation of treatment. Median time of onset of adverse effects was around 4.5 months. This timeframe contrasts with a previous study, where cases of azathioprine-induced hepatotoxicity manifested much earlier, with a median onset time of 14 days.^25^ The dog in our study did not receive any concurrent treatment that could have caused hepatotoxicity; azathioprine was then incriminated. The hepatic toxicity observed in azathioprine did not appear to be dose-dependent, as the majority of dogs received the same dosage of azathioprine as the 1 experiencing hepatotoxicity. In previous investigations involving the utilization of azathioprine for the management of diverse autoimmune disorders, the occurrence of hepatotoxicity was not detected.^20^^,^^21^

In this study, cases of optic neuritis were included when they were associated with intra-axial lesions on MRI, supporting the diagnosis of MUO. Therefore, cases presenting with isolated optic neuritis were not included, in line with recent literature where such cases were also not reported. We also included cases with myelopathy only, which is in contrast with previous studies. Interestingly, in the present study, the median survival time of these dogs was not significantly longer than that of dogs presenting with encephalopathy. This suggests that, despite showing only clinical signs of myelopathy, the disease might be more diffusely distributed within the CNS than previously assumed. Notably, 2 cases in this study cohort exhibited multifocal lesions in both spinal cord and forebrain. It is not clear whether other cases might have exhibited inflammatory lesions elsewhere in the CNS, as the MRI examination is often limited to 1 or 2 regions to limit its duration.

Non regenerative anemia was the most common adverse effect identified in this study. The delayed occurrence of cytopenia, as observed in the present study, aligns with the findings of the previously mentioned study.^25^ Azathioprine can induce myelosuppression, particularly during the initial stages of treatment. However, it is typically characterized by thrombocytopenia or neutropenia preceding anemia, which contrasts with this study results. In all cases except 1, myelosuppression was mild and reversible. For this 1 dog, the severity of anemia surpassed that observed in the other animals. Deterioration of anemia was fast and developed in 10 days until the dog died from this adverse effect. It is believed that in instances where the myelosuppression goes unrecognized, there is a likelihood of progressive development of severe myelofibrosis, which subsequently leads to a guarded prognosis.^26^ In our case, anemia was identified but worsened even after discontinuation of azathioprine. Based on the findings of this study and previous investigations, we recommend conducting frequent complete blood counts during treatment, to allow early detection of potential myelosuppression.

In the present study, dogs exhibiting seizures did not have significantly shorter survival times compared to those without. Additionally, survival time was not significantly longer in dogs without relapse compared to those that experienced relapse. These results contrast with previous study on association of prednisolone and azathioprine in treating MUO.^18^^,^^27^ In the present study, several cases were euthanized because of refractory status epilepticus or died during status. The lack of a significant difference between the 2 groups in this study does not rule out a difference in the overall study population. Due to the cases variability and the size of the study, the 95% CI of median survival times are quite large and therefore limit their clinical relevance. In Figure 3, we included cases that were slated to receive the combination but died before receiving azathioprine. Statistical analysis in this study was considered exploratory, as no sample size calculation was performed and the expected effect sizes were unknown, precluding definitive hypothesis testing. Another limit relies on its retrospective nature. Variations in treatment, including dosage and duration, were noted based on the clinician habits and owners’ compliance. Some dogs in our study were lost to follow up and prevented us from drawing conclusion regarding long-term treatment adverse effects in those cases.

### Conclusion

The results indicate that the combination of azathioprine and prednisolone is associated with long median survival times for dogs with MUO. Adverse drug reactions were reversible in most cases but can be fatal in a minority of cases (about 4%). Therefore, hematological values and hepatic variables should be monitored closely during treatment.

## Supplementary Material

aalaf002_Supplemental_Files

## Off-label antimicrobial declaration

Authors declare no off-label use of antimicrobials.

## Institutional animal care and use committee or other approval declaration

Authors declare no institutional animal care and use committee or other approval was needed.

## Abbreviations

DLA: dog leukocyte antigen
GME: granulomatous meningoencephalitis
MRI: magnetic resonance imaging
MUO: meningoencephalomyelitis
NLE: necrotizing leucoencephalitis
NME: necrotizing meningoencephalomyelitis
PCR: polymerase chain reaction
TNCC: total nucleated cell count
