# Supplementary material for: Combination of azathioprine and prednisolone as a treatment for meningoencephalomyelitis of unknown origin in dogs: 54 cases
Source: J Vet Intern Med. 2026 Jan 21;40(1):aalaf002. doi: 10.1093/jvimsj/aalaf002 (PMC12881960; doi:10.1093/jvimsj/aalaf002)
Supplement: aalaf002_Supplemental_Files [file aalaf002_supplemental_files.zip › Supplementary_Material_Figure S1.pdf]

Supplementary Figure 1: Histogram showing time of onset of adverse effects among the 11 dogs that exhibited adverse reaction.

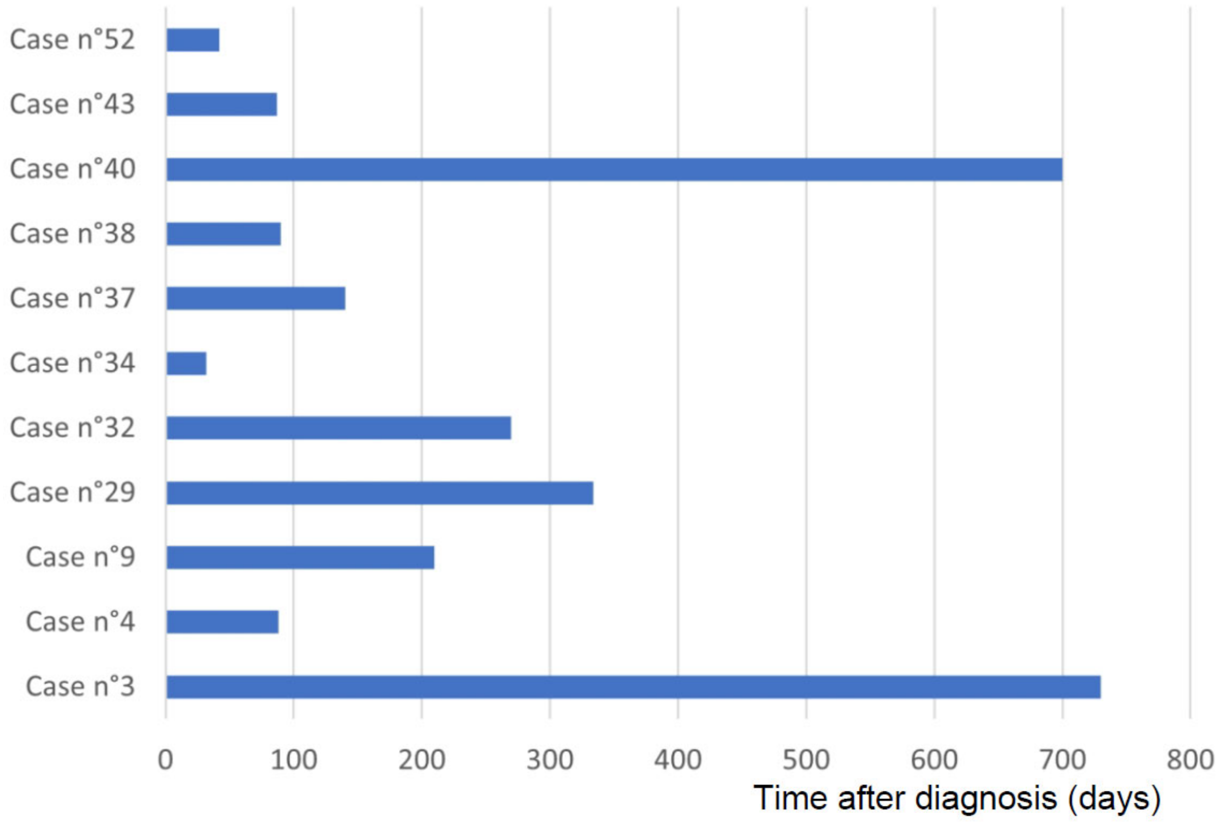

Supplementary Table 1: Supplementary data regarding the cases included (that received the combination azathioprine and prednisolone, and the cases identified in intention to treat)
